# Accuracy of automated computer-aided risk scoring systems to estimate the risk of COVID-19: a retrospective cohort study

**DOI:** 10.1186/s13104-024-06773-0

**Published:** 2024-04-18

**Authors:** Muhammad Faisal, Mohammed Amin Mohammed, Donald Richardson, Massimo Fiori, Kevin Beatson

**Affiliations:** 1 Centre for Digital Innovations in Health & Social Care, Faculty of Health Studies, University of Bradford, Bradford, UK; 2 Wolfson Centre for Applied Health Research, Bradford, UK; 3 Faculty of Health Studies, University of Bradford, Richmond Road, BD7 1DP Bradford, UK; 4 NHS Midlands and Lancashire Commissioning Support Unit, The Strategy Unit, Kingston House, B70 9LD West Bromwich, UK; 5 Consultant Renal Physician York & Scarborough Teaching Hospitals NHS Foundation Trust, York, UK; 6 York & Scarborough Teaching Hospitals NHS Foundation Trust, York, UK

**Keywords:** National early warning score, COVID-19, Mortality risk, Computer-aided risk scoring systems

## Abstract

**Background:**

In the UK National Health Service (NHS), the patient’s vital signs are monitored and summarised into a National Early Warning Score (NEWS) score. A set of computer-aided risk scoring systems (CARSS) was developed and validated for predicting in-hospital mortality and sepsis in unplanned admission to hospital using NEWS and routine blood tests results. We sought to assess the accuracy of these models to predict the risk of COVID-19 in unplanned admissions during the first phase of the pandemic.

**Methods:**

Adult ( > = 18 years) non-elective admissions discharged (alive/deceased) between 11-March-2020 to 13-June-2020 from two acute hospitals with an index NEWS electronically recorded within ± 24 h of admission. We identified COVID-19 admission based on ICD-10 code ‘U071’ which was determined by COVID-19 swab test results (hospital or community). We assessed the performance of CARSS (CARS_N, CARS_NB, CARM_N, CARM_NB) for predicting the risk of COVID-19 in terms of discrimination (c-statistic) and calibration (graphically).

**Results:**

The risk of in-hospital mortality following emergency medical admission was 8.4% (500/6444) and 9.6% (620/6444) had a diagnosis of COVID-19. For predicting COVID-19 admissions, the CARS_N model had the highest discrimination 0.73 (0.71 to 0.75) and calibration slope 0.81 (0.72 to 0.89) compared to other CARSS models: CARM_N (discrimination:0.68 (0.66 to 0.70) and calibration slope 0.47 (0.41 to 0.54)), CARM_NB (discrimination:0.68 (0.65 to 0.70) and calibration slope 0.37 (0.31 to 0.43)), and CARS_NB (discrimination:0.68 (0.66 to 0.70) and calibration slope 0.56 (0.47 to 0.64)).

**Conclusions:**

The CARS_N model is reasonably accurate for predicting the risk of COVID-19. It may be clinically useful as an early warning system at the time of admission especially to triage large numbers of unplanned admissions because it requires no additional data collection and is readily automated.

**Supplementary Information:**

The online version contains supplementary material available at 10.1186/s13104-024-06773-0.

## Introduction

The novel coronavirus SARS-CoV-2, which was declared as a pandemic on 11-March 2020, which produces the newly identified disease ‘COVID-19’ in patients with symptoms (Coronaviridae Study Group of the International Committee on Taxonomy of Viruses [1]), has challenged health care systems worldwide.

Patients with COVID-19 admitted to a hospital can develop severe disease with life-threatening respiratory and/or multi-organ failure [2, 3] with a high risk of mortality. It is recommended that patients at risk of deterioration are referred to critical care. The appropriate early assessment and management of patients with COVID-19 are important in ensuring high-quality care [4, 5].

In the UK National Health Service (NHS), the patient’s vital signs are monitored and summarised into a National Early Warning Score (NEWS) [6]. NEWS is calculated from six physiological variables or vital signs—respiration rate, oxygen saturation, temperature, systolic blood pressure, heart rate and level of consciousness (alert, voice, pain, unresponsive) and use of supplemental oxygen. NEWS points are allocated according to clinical observations (see Table S1).

We have developed four automated, computer-aided risk scores to predict the patient’s risk of mortality (CARM_N & CARM_NB) and sepsis (CARS_N & CARS_NB) following emergency medical admission to hospital [7–10]. The _N models use NEWS and the _NB models incorporate routine blood test results. We refer to this suite of risk equations as computer-aided risk scoring systems (CARSS).

Our aim in this study was to assess the accuracy of CARSS in predicting the risk of COVID-19 in unplanned admissions to a teaching hospital during the first phase of the novel coronavirus SARS CoV-2 (COVID-19) pandemic. We are not developing new risk prediction models, we are assessing the performance of existing models, re-purposed for COVID-19.

## Methods

### Setting & data

Our cohort of unplanned admissions is from two acute hospitals which are approximately 65 km apart in the Yorkshire & Humberside region of England—Scarborough hospital (*n* ∼ 300 beds) and York Hospital (YH) (*n* ∼ 700 beds), managed by York Teaching Hospitals NHS Foundation Trust. For this study, the two acute hospitals are combined into a single dataset and analysed collectively. The hospitals have electronic NEWS scores and vital signs recording which is routinely collected as part of the patient’s process of care (see Table S1).

We considered all adult (age ≥ 18 years) emergency medical admissions (excluding ambulatory care area patients), discharged (alive/deceased) during 3 months (11 March 2020 to 13 June 2020), with electronic NEWS recorded within ± 24 h of admission. This on-admission NEWS score is referred to as the index NEWS.

For each emergency admission, we obtained a pseudonymised patient identifier, patient’s age (years), gender (male/female), discharge status (alive/dead), admission and discharge date and time, diagnoses codes based on the 10th revision of the International Statistical Classification of Diseases (ICD-10) [11, 12], NEWS (including its subcomponents respiratory rate [breaths per minute], temperature [^o^C], systolic pressure [mmHg], pulse rate [beats per minute], oxygen saturation [percentage], oxygen supplementation [yes/no], and alertness level [alert, voice, pain, unconscious] ) [6, 13], blood test results (albumin [g/L], creatinine [umol/L], haemoglobin [g/l], potassium [mmol/L], sodium [mmol/L], urea [mmol/L], and white cell count [10^9^ cells/L]), and Acute Kidney Injury (AKI) score.

**Table 1:** Four risk scores for predicting the risk of mortality and sepsis, known as computer-aided risk scoring systems (CARSS)

Computer-Aided Risk (CAR) score	NEWS data only (N)	NEWS and Blood test results data (NB)
**Mortality (M)**	CARM_N	CARM_NB
**Sepsis (S)**	CARS_N	CARS_NB

We had developed and externally validated four risk scores: (1) CARM_N for predicting in-hospital mortality based on NEWS [10]; (2) CARM_NB for predicting in-hospital mortality that incorporates routine blood test results [7]; CARS_N for predicting sepsis based on NEWS [9]; CARS_NB for predicting sepsis that incorporates routine blood test results [8] (see Table 1). These four equations are collectively known as computer-aided risk scoring systems (CARSS), calculated using index NEWS and blood test results. We excluded records where the index NEWS (or blood test results) was not within ± 24 h (± 96 h) or was missing/not recorded at all (see Table S2).

The ICD-10 code ‘U071’ was used to identify records with COVID-19. We searched primary and secondary ICD-10 codes for ‘U071’ for identifying COVID-19. We also linked positive laboratory results for COVID-19 swabs to an automated diagnostic coding entry in the patient electronic health record.

### Statistical analyses

We report discrimination and calibration statistics as performance measures for CARSS [14].

We determined the discrimination of CARSS using the concordance statistic (c-statistic) that gives the probability of randomly selected patients who experienced COVID-19 had a higher risk score than a Non-Covid-19 patient. For a binary outcome (COIVD-19/Non-Covid-19), the c-statistic is the area under the Receiver Operating Characteristics (ROC) curve [15]. The ROC curve is a plot of the sensitivity, (true positive rate), versus 1-specificity, (false positive rate), for consecutive predicted risks. A c-statistic of 0.5 is no better than tossing a coin, whilst a perfect model has a c-statistic of 1. In general, values less than 0.7 are considered to show poor discrimination, values of 0.7 to 0.8 can be described as reasonable, and values above 0.8 suggest good discrimination [16].

Calibration measures a model’s ability to generate predictions that are on average close to the average observed outcome and can be readily seen on a scatter plot (y-axis observed risk, x-axis predicted risk). Perfect predictions should be on the 45° line. We internally validated and assessed the calibration for all the models using the bootstrapping approach [17, 18]. The overall statistical performance was assessed using the scaled Brier score which incorporates both discrimination and calibration [14]. The Brier score is the squared difference between actual outcomes and predicted risk of COVID-19, scaled by the maximum Brier score such that the scaled Brier score ranges from 0 to 100%. Higher values indicate superior models. The 95% confidence interval for the scaled Brier score was calculated using bootstrap approach [19].

We followed the STROBE guidelines to report the findings [20]. All analyses were undertaken using *R* [21] and Stata [22]. The 95% confidence interval for the c-statistic was computed using DeLong’s method as implemented in the *pROC* library [23].

## Results

### Cohort description

There were 6480 discharges over 3 months. We excluded 36 (0.6%) records because the index NEWS was not recorded within ± 24 h of the admission date/time or NEWS was missing or not recorded at all (see Table S2). We further excluded 1175 (18.1%) because absence of blood test results.

The prevalence of COVID-19 was 9.6% (620/6444) and of these 32% (199/620) deceased at discharge. The demographic, vital signs and outcome profiles of the COVID-19 versus non-COVID-19 admissions and discharge deceased versus discharged alive are shown in Table 2 and Figure S1-S2. COVID-19 admissions were older (73.3 vs. 67.7, *p* < 0.001), more likely to be male (54.7% vs. 50.1%, *p* < 0.001), with higher index NEWS (4.0 vs. 2.5, *p* < 0.001). They also had longer hospital stay (7.3 days vs. 3.0 days, *p* < 0.001) and higher in-hospital mortality (32.1% vs. 5.8%, *p* < 0.001). The average CARSS (CARM_N, CARM_NB, CARS_N, CARS_NB) risk was generally higher for COVID-19 admissions and for those who were deceased at discharge.

### Statistical modelling results

**Fig. 1:**
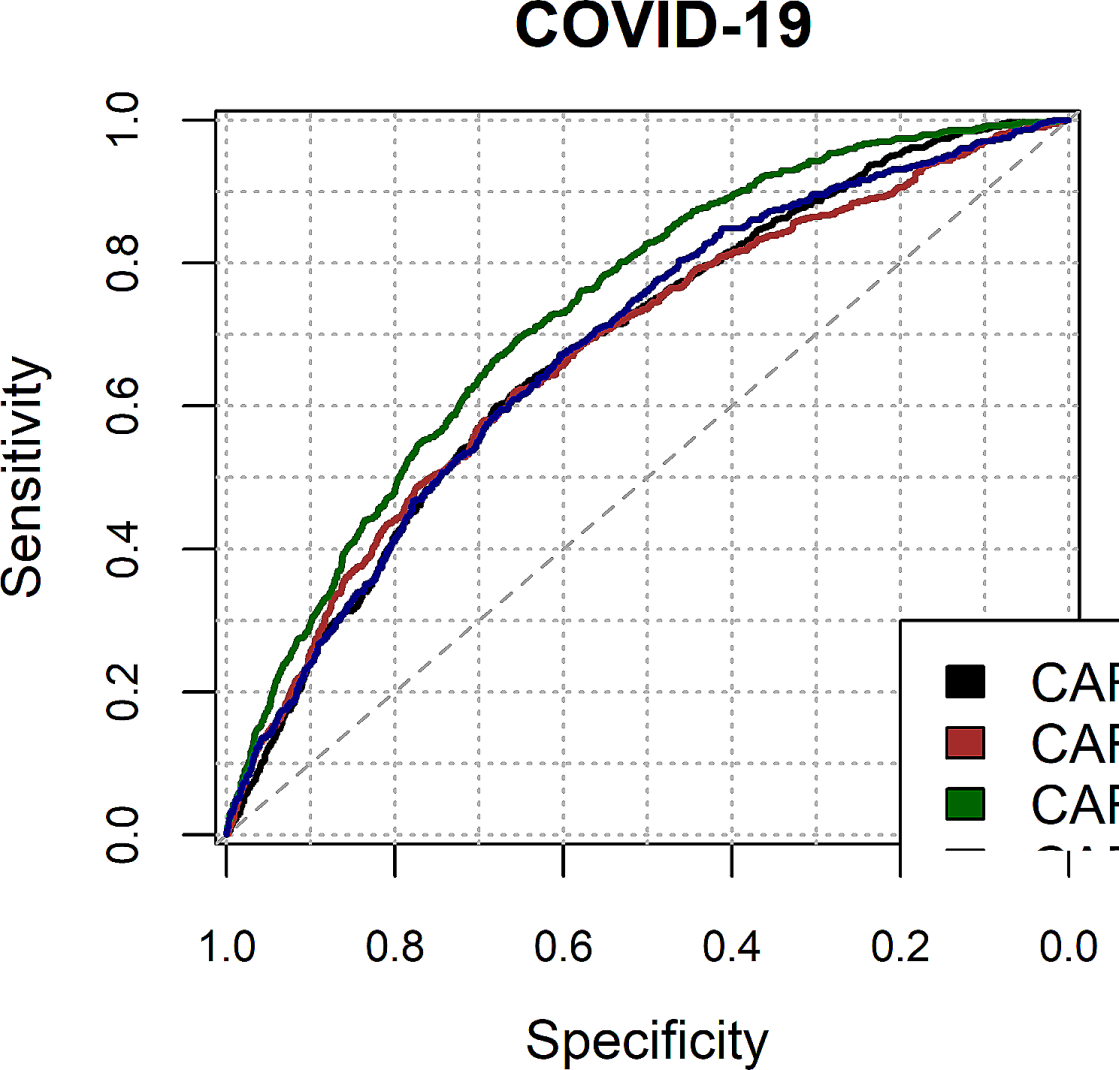
Receiver Operating Characteristic curve for four CARSS models in predicting the risk of COVID-19. CARM_N: for predicting mortality with NEWS data only; CARM_NB: for predicting mortality with NEWS and Blood test results data; CARS_N: for predicting sepsis with NEWS data only; CARS_NB: for predicting sepsis with NEWS and Blood test results data

**Fig. 2:**
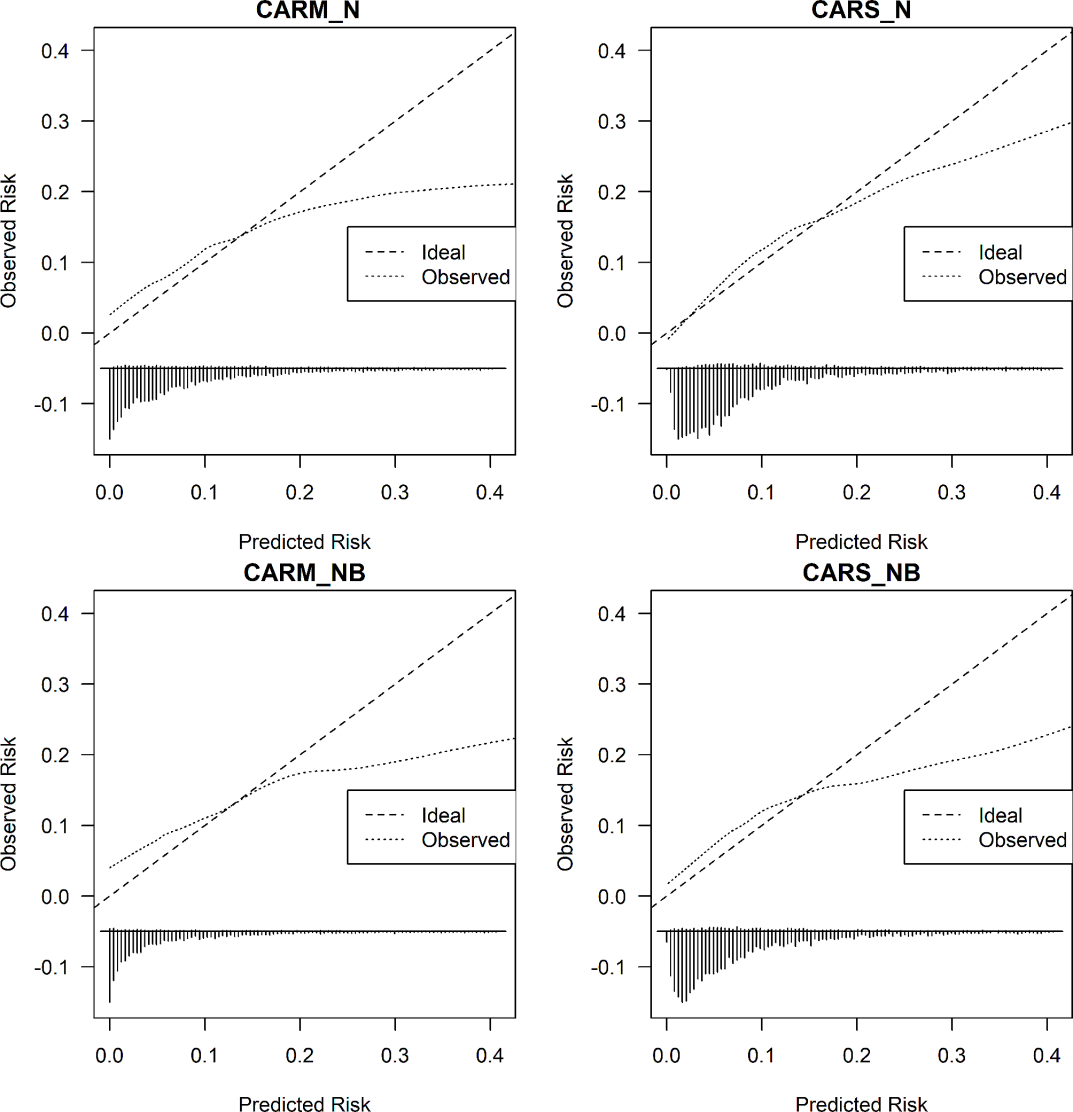
External validation of CARSS models, respectively for predicting the risk of COVID-19. NB: We limit the risk of COVID-19 to 0.40 for visualisation purpose because beyond this point, we have few patients

We assessed the four CARSS models (CARM_N, CARM_NB, CARS_N, CARS_NB) performance according to discrimination (c-statistic) and calibration (graphically) in predicting the risk of COVID-19 (see Table 3; Figs. 1 and 2).

For predicting COVID-19 admissions, the CARS_N model performed better than others in terms of discrimination 0.73 (95%CI 0.71 to 0.75) and calibration slope 0.81 (95%CI 0.72 to 0.89) compared to other CARSS models: CARM_N (discrimination: 0.68 (0.66 to 0.70) and calibration slope 0.47 (0.41 to 0.54)), CARM_NB (discrimination: 0.68 (0.65 to 0.70) and calibration slope 0.37 (0.31 to 0.43)), and CARS_NB (discrimination: 0.68 (0.66 to 0.70) and calibration slope 0.56 (0.47 to 0.64)).

**Table 2:** Characteristics of emergency medical admissions in COVID-19 versus non-COVID-19 who discharged alive/deceased

Characteristic	COVID-19		Non-COVID-19	
	Discharged Deceased	Discharged Alive	Discharged Deceased	Discharged Alive
N	199	421	336	5488
Median Length of Stay (IQR)	9.61 (14.43)	6.73 (10.52)	4.72 (8.88)	2.96 (5.28)
Male (%)	123 (61.81)	216 (51.31)	169 (50.3)	2749 (50.09)
Mean Age [years] (SD)	80.22 (10.01)	70.08 (16.43)	79.44 (12.65)	67.02 (19.14)
Mean NEWS (SD)	4.94 (3.02)	3.52 (2.5)	4.89 (3.42)	2.33 (2.08)
Mean CARM_N (SD)	0.14 (0.11)	0.06 (0.07)	0.15 (0.13)	0.04 (0.06)
Mean CARM_NB (SD)	0.15 (0.15)	0.06 (0.08)	0.16 (0.17)	0.04 (0.06)
Mean CARS_N (SD)	0.36 (0.19)	0.25 (0.16)	0.28 (0.16)	0.16 (0.13)
Mean CARS_NB (SD)	0.34 (0.2)	0.21 (0.16)	0.29 (0.18)	0.15 (0.13)

* Blood test results are missing 1175 (18.1%)

**Table 3:** Performance of CARSS models for predicting the risk of COVID-19

Model	Mean risk without adverse outcome	Mean risk with adverse outcome	Absolute risk difference	Scaled brier score	Discrimination AUC (95% CI)	Calibration Slope (95% CI)
CARM_N	0.09	0.15	0.06	-0.02 (-0.03 to -0.01)	0.68 (0.66 to 0.70)	0.47 (0.41 to 0.54)
CARM_NB	0.09	0.17	0.08	-0.05 (-0.06 to -0.04)	0.68 (0.65 to 0.70)	0.37 (0.31 to 0.43)
CARS_N	0.09	0.17	0.08	0.05 (0.04 to 0.06)	0.73 (0.71 to 0.75)	0.81 (0.72 to 0.89)
CARS_NB	0.09	0.16	0.07	0.01 (0.00 to 0.02)	0.68 (0.66 to 0.70)	0.56 (0.47 to 0.64)

CARM_N: for predicting mortality with NEWS data only; CARM_NB: for predicting mortality with NEWS and Blood test results data; CARS_N: for predicting sepsis with NEWS data only; CARS_NB: for predicting sepsis with NEWS and Blood test results data

## Discussion

We assessed the performance of four computer-aided risk scores to predict the risk of COVID-19 in unplanned admissions to hospital. We found that the CARS_N model for sepsis (based on NEWS) had the best performance for predicting the risk of COVID-19. CARS_N was developed for predicting sepsis and we found it has good discrimination and calibration compared to other CARSS models. This may reflect the reported overlap in features between sepsis and COVID-19, such as hyper inflammation and coagulopathy which also contribute to disease severity and death in COVID-19 patients [24]. Zhou et al. [25] found that the Sequential Organ Failure Assessment (SOFA) score (for sepsis) is associated with in-hospital mortality in COVID-19 patients.

A recent systematic review identified models to predict mortality from COVID-19 with c-statistics that ranged from 0.87 to 1 [26]. However, despite these high c-statistics, the review authors cautioned against the use of these models in clinical practice because of the high risk of bias and poor reporting of studies which are likely to have led to optimistic results [26]. The main advantages of our models are that they are (1) rigorously developed and externally validated, (2) designed to incorporate data which are already available in the patient’s electronic health record thus place no additional data collection or computational burden on clinicians and (3) are readily automated. The CARS_N model is particularly attractive because it uses NEWS data which can be available within a short while (< 30 min) of admission and so can support early clinical decision making about patients, which is essential to ensuring safe, high quality care.

There are several limitations to our study: (1) This data is from a single NHS Trust, and to understand the extent to which these findings are generalisable, further study is required (2) We used the index NEWS and blood test results which reflects the ‘on-admission’ risk of mortality of the patients. Nonetheless, NEWS and blood test results are repeatedly updated for each patient according to local hospital protocols (Figure S5 in supplementary material) (3) We identified COVID-19 based on ICD-10 code ‘U071’ which was determined by COVID-19 swab test results (hospital or community) and clinical judgment and so our findings are constrained by the accuracy of these methods [27, 28] (4) We have used NEWS in our data but since the NEWS2 is now widely used, further study is required to determine the accuracy of NEWS2 based models [29].

## Conclusion

The CARS_N model is reasonably accurate for predicting the risk of COVID-19. It may be clinically useful as an early warning system at the time of admission especially to triage large numbers of unplanned admissions because it requires no additional data collection and is readily automated.

### Electronic supplementary material

Below is the link to the electronic supplementary material.

Supplementary Material 1

## Data Availability

The data that support the findings of this study are available from NHS York hospital trust but restrictions apply to the availability of these data, which were used under license for the current study, and so are not publicly available. However, if anyone is interested in the data, then they should contact the R&D offices in the first instance https://www.research.yorkhospitals.nhs.uk/about-us1/our-directorates/.
