# Supplementary material for: Accuracy of automated computer-aided risk scoring systems to estimate the risk of COVID-19: a retrospective cohort study
Source: BMC Res Notes. 2024 Apr 18;17:109. doi: 10.1186/s13104-024-06773-0 (PMC11027522; doi:10.1186/s13104-024-06773-0)
Supplement: Supplementary file 1 — Supplementary Material 1 [file 13104_2024_6773_MOESM1_ESM.pdf]

Supplementary Material

Physiological Parameters	3	2	1	0	1	2	3
**Respiration Rate**	≤8		9 - 11	12 - 20		21 - 24	≥25
**Oxygen Saturations**	≤91	92 - 93	94 - 95	≥96			
**Any Supplemental Oxygen**		Yes		No			
**Temperature**	≤35.0		35.1 - 36.0	36.1 - 38.0	38.1 - 39.0	≥39.1	
**Systolic BP**	≤90	91 - 100	101 - 110	111 - 219			≥220
**Heart Rate**	≤40		41 - 50	51-90	91 - 110	111 - 130	≥131
**Level of Consciousness**				Alert			Voice, Pain, or Unconscious

Table S1: NEWS scoring chart

The NEWS [https://www.rcplondon.ac.uk/projects/outputs/national-early-warning-score-news] is based on a scoring system in which a score is allocated to vital signs physiological measurements already undertaken when patients present to or are being monitored in hospital. A score is allocated to each as they are measured, the magnitude of the score reflecting how extreme the parameter varies from the norm. This score is then aggregated and uplifted for people requiring oxygen.

Characteristic	COVID-19	Non-COVID-19	All
	N (%)	N (%)	N (%)
Total emergency medical discharges between 11 Mar 20 to 13 June 20	622	5858	6480
Excluded: No NEWS recorded (%)	0 (0.0)	19 (0.3)	19 (0.3)
Excluded: First NEWS after 24 hours of admission (%)	2 (0.3)	15 (0.3)	17 (0.3)
Excluded: No or missing blood test results recorded (%)	111 (17.8)	1064 (18.1)	1175 (18.1)
Total excluded (%)	113 (18.2)	1098 (18.9)	1211 (18.7)
Total included (%)	509 (81.8)	4760 (81.1)	5269 (81.3)

Table S2 Number of emergency medical admissions included/excluded

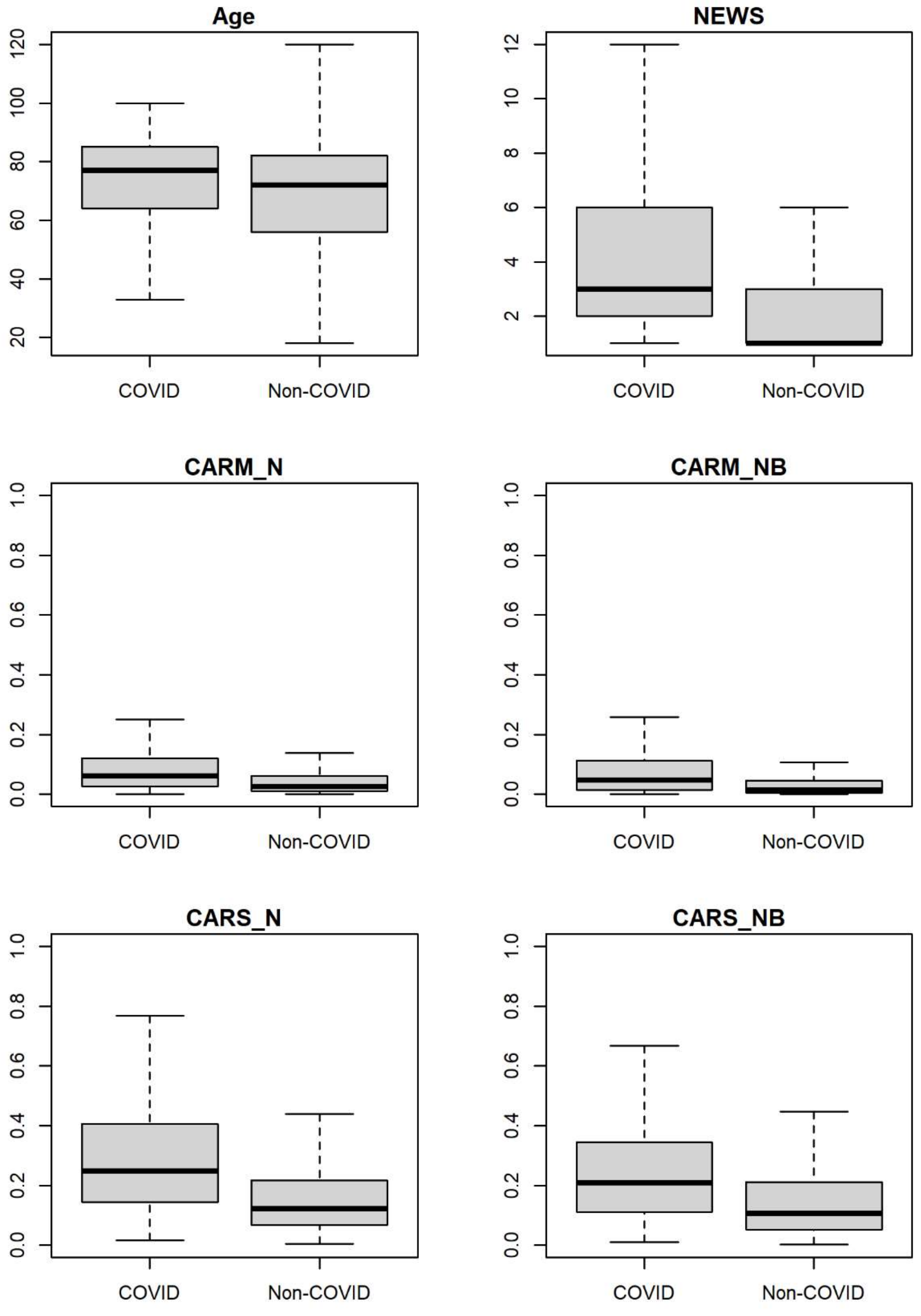

Figure S1 Boxplot for age, NEWS and CARSS without outliers with respect to COVID-19 status (COVID/Non-COVID)

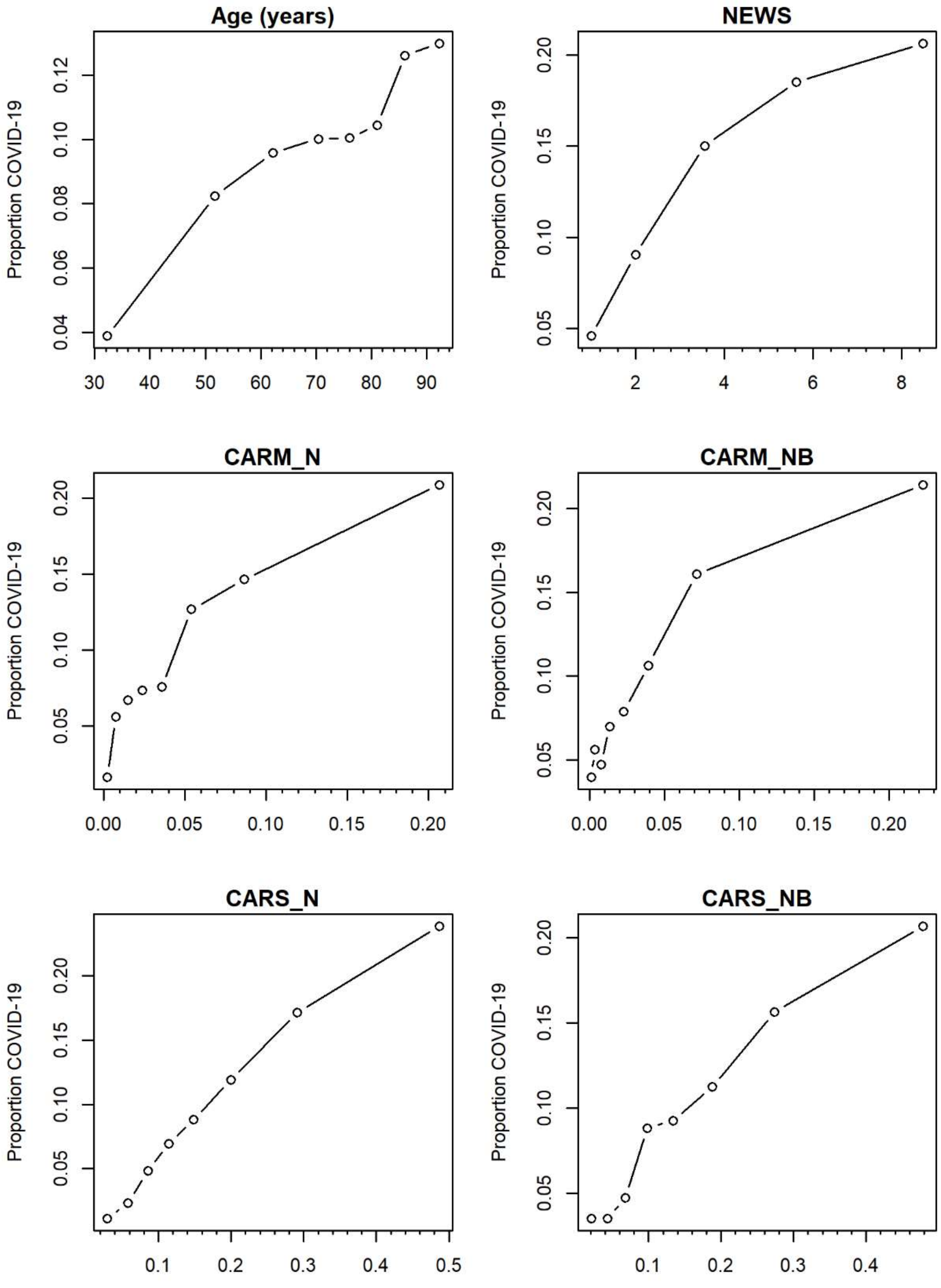

Figure S2 Scatter plots showing the observed risk of COVID-19 with age, NEWS, and CARSS

NB: y-axis range changes in each plot.

```
#######R CODE FOR ANALYSIS #########
#######

myperf <- function (pred = pred, actual=actual, heading="modelname",outline=FALSE){

  disc_measure <- tapply(pred, actual, mean)
  slope0=disc_measure[1]
  slope1=disc_measure[2]
  slope=disc_measure[2]-disc_measure[1]

  brier_scaled <- brier.scaled(pred = pred, actual = actual)$Bscaled

  auc0 <- ci.auc(actual,pred)[1]
  auc <- ci.auc(actual,pred)[2]
  auc1 <- ci.auc(actual,pred)[3]

  boxplot(pred ~ actual, ylim = c(0,1),outline=outline,xlab="",ylab="Predicted Risk")
  btitle <- paste("AUC: ", round(auc,2))
  ctitle <- paste("SLOPE: ", round(slope,2))
  ftitle <- paste(heading,btitle,ctitle)
  title(ftitle)
  points(disc_measure,col="red",pch=18)

  return(c(slope0,slope1,slope, brier_scaled,auc0,auc,auc1))

}


df11 <- df[df$covid==1 & df$died==1, ]

av11 <- rbind(
  length(df11$covid),
  median(df11$los),
  as.numeric(table(df11$male)[2]),
  mean(df11$age),
  mean(df11$calc_news),
  mean(df11$carm_n),
  mean(df11$carm_nb,na.rm=TRUE),
  mean(df11$cars_n),
  mean(df11$cars_nb,na.rm=TRUE)

)


sd11 <- rbind(
  0,
  as.numeric(quantile(df11$los,0.75) - quantile(df11$los,0.25)),
  as.numeric(100*(table(df11$male)/length(df11$male))[2]),
  sd(df11$age),
  sd(df11$calc_news),
  sd(df11$carm_n),
  sd(df11$carm_nb,na.rm=TRUE),
  sd(df11$cars_n),
  sd(df11$cars_nb,na.rm=TRUE)

)




df10 <- df[df$covid==1 & df$died==0, ]

av10 <- rbind(
  length(df10$covid),
  median(df10$los),
  as.numeric(table(df10$male)[2]),
  mean(df10$age),
  mean(df10$calc_news),
  mean(df10$carm_n),
  mean(df10$carm_nb,na.rm=TRUE),
  mean(df10$cars_n),
```

```
  mean(df10$cars_nb,na.rm=TRUE)

)

sd10 <- rbind(
  0,
  as.numeric(quantile(df10$los,0.75) - quantile(df10$los,0.25)),
  as.numeric(100*(table(df10$male)/length(df10$male))[2]),
  sd(df10$age),
  sd(df10$calc_news),
  sd(df10$carm_n),
  sd(df10$carm_nb,na.rm=TRUE),
  sd(df10$cars_n),
  sd(df10$cars_nb,na.rm=TRUE)

)

df01 <- df[df$covid==0 & df$died==1, ]

av01 <- rbind(
  length(df01$covid),
  median(df01$los),
  as.numeric(table(df01$male)[2]),
  mean(df01$age),
  mean(df01$calc_news),
  mean(df01$carm_n),
  mean(df01$carm_nb,na.rm=TRUE),
  mean(df01$cars_n),
  mean(df01$cars_nb,na.rm=TRUE)

)

sd01 <- rbind(
  0,
  as.numeric(quantile(df01$los,0.75) - quantile(df01$los,0.25)),
  as.numeric(100*(table(df01$male)/length(df01$male))[2]),
  sd(df01$age),
  sd(df01$calc_news),
  sd(df01$carm_n),
  sd(df01$carm_nb,na.rm=TRUE),
  sd(df01$cars_n),
  sd(df01$cars_nb,na.rm=TRUE)

)

df00 <- df[df$covid==0 & df$died==0,]

av00 <- rbind(
  length(df00$covid),
  median(df00$los),
  as.numeric(table(df00$male)[2]),
  mean(df00$age),
  mean(df00$calc_news),
  mean(df00$carm_n),
  mean(df00$carm_nb,na.rm=TRUE),
  mean(df00$cars_n),
  mean(df00$cars_nb,na.rm=TRUE)

)


sd00 <- rbind(
  0,
  as.numeric(quantile(df00$los,0.75) - quantile(df00$los,0.25)),
  as.numeric(100*(table(df00$male)/length(df00$male))[2]),
  sd(df00$age),
  sd(df00$calc_news),
  sd(df00$carm_n),
  sd(df00$carm_nb,na.rm=TRUE),
  sd(df00$cars_n),
  sd(df00$cars_nb,na.rm=TRUE)

)
```

```
res <- cbind(av11,sd11, av10,sd10,av01,sd01, av00,sd00)
row.names(res)=c("N","los","male","age","NEWS2","carm_n","carmn_nb","cars_n","cars_nb")


write.csv(res,"table1.csv")



tiff("figS1.tiff", height = 8, width = 6, units = 'in', res=300)

par(mfrow=c(3,2), mar=c(4,4,1.5,1.5), oma=c(1.5,2,1,1))
df$covidf <- factor(df$covid, labels= c("Non-COVID", "COVID"))
df$covidf <- factor(df$covidf, levels(df$covidf)[c(2,1)])

df1$covidf <- factor(df1$covid, labels= c("Non-COVID", "COVID"))
df1$covidf <- factor(df1$covidf, levels(df1$covidf)[c(2,1)])

boxplot(df$age~df$covidf,outline=FALSE,xlab="",ylab="");title("Age")
boxplot(df$calc_news~df$covidf,outline=FALSE,xlab="",ylab="");title("NEWS")

boxplot(df$carm_n~df$covidf,outline=FALSE,xlab="",ylab="",ylim=c(0,1));title("CARM_N")
boxplot(df1$carm_nb~df1$covidf,outline=FALSE,xlab="",ylab="",ylim=c(0,1));title("CARM_NB")
boxplot(df$cars_n~df$covidf,outline=FALSE,xlab="",ylab="",ylim=c(0,1));title("CARS_N")
boxplot(df1$cars_nb~df1$covidf,outline=FALSE,xlab="",ylab="",ylim=c(0,1));title("CARS_NB")
#boxplot(df$calc_news~df$covidf,outline=FALSE,xlab="",ylab="",ylim=c(0,16));title("NEWS")
#boxplot(df$NEWS~df$covidf,outline=FALSE,xlab="",ylab="",ylim=c(0,16));title("NEWS2")

dev.off()




tiff("FigS2.tiff", height = 8, width = 6, units = 'in', res=300)

par(mfrow=c(3,2), mar=c(4,4,1.5,1.5), oma=c(1.5,2,1,1))
library(Hmisc)

x <- cut2(df$age, levels.mean=TRUE, g=8)
y <- tapply(df$covid, x, mean)
plot(levels(x), y, main="Age (years)", xlab="", ylab="Proportion COVID-19", type='b') #ylim=c(0,0.6))
minor.tick(nx=5, ny=0, tick.ratio=0.3)


x <- cut2(df$calc_news, levels.mean=TRUE, g=8)
y <- tapply(df$covid, x, mean)
plot(levels(x), y, main="NEWS", xlab="", ylab="Proportion COVID-19", type='b') #ylim=c(0,0.6))
minor.tick(nx=5, ny=0, tick.ratio=0.3)

x <- cut2(df$carm_n, levels.mean=TRUE, g=8)
y <- tapply(df$covid, x, mean)
plot(levels(x), y, main="CARM_N", xlab="", ylab="Proportion COVID-19", type='b') #ylim=c(0,0.6))
minor.tick(nx=5, ny=0, tick.ratio=0.3)

x <- cut2(df$carm_nb, levels.mean=TRUE, g=8)
y <- tapply(df$covid, x, mean)
plot(levels(x), y, main="CARM_NB", xlab="", ylab="Proportion COVID-19", type='b') #ylim=c(0,0.6))
minor.tick(nx=5, ny=0, tick.ratio=0.3)

x <- cut2(df$cars_n, levels.mean=TRUE, g=8)
y <- tapply(df$covid, x, mean)
plot(levels(x), y, main="CARS_N", xlab="", ylab="Proportion COVID-19", type='b') #ylim=c(0,0.6))
minor.tick(nx=5, ny=0, tick.ratio=0.3)

x <- cut2(df$cars_nb, levels.mean=TRUE, g=8)
y <- tapply(df$covid, x, mean)
plot(levels(x), y, main="CARS_NB", xlab="", ylab="Proportion COVID-19", type='b') #ylim=c(0,0.6))
minor.tick(nx=5, ny=0, tick.ratio=0.3)

dev.off()
```

```
df$lcarm_n2 <- df$lcarm_n+0.52
df$pmn1 = exp(df$lcarm_n2)/(1+exp(df$lcarm_n2))

df1$lcarm_nb2 <- df1$lcarm_nb+0.74
df1$pmnb1 = exp(df1$lcarm_nb2)/(1+exp(df1$lcarm_nb2))


df$lcars_n2 <- df$lcars_n-0.98
df$pmn2 = exp(df$lcars_n2)/(1+exp(df$lcars_n2))

df1$lcars_nb2 <- df1$lcars_nb-0.87
df1$pmnb2 = exp(df1$lcars_nb2)/(1+exp(df1$lcars_nb2))
df$lcarm_n2c <- df$lcarm_n+0.71
df$pcn1 = exp(df$lcarm_n2c)/(1+exp(df$lcarm_n2c))

df1$lcarm_nb2c <- df1$lcarm_nb+0.93
df1$pcnb1 = exp(df1$lcarm_nb2c)/(1+exp(df1$lcarm_nb2c))


df$lcars_n2c <- df$lcars_n-0.80
df$pcn2 = exp(df$lcars_n2c)/(1+exp(df$lcars_n2c))

df1$lcars_nb2c <- df1$lcars_nb-0.70
df1$pcnb2 = exp(df1$lcars_nb2c)/(1+exp(df1$lcars_nb2c))


df$flag_news=0
df$flag_news[df$calc_news>=5]=1

df$flag_news2=0
df$flag_news2[df$NEWS>=5]=1

#covid cars_n is now pcn1
#covid cars_nb is now pcnb1
#covid cars_n is now pcn2
#covid cars_nb is now pcnb2



c1 <- myperf(df$pcn1, df$covid, heading ="In-hospital",outline=TRUE)
c2 <- myperf(df1$pcnb1, df1$covid, heading ="In-hospital",outline=TRUE)
c3 <- myperf(df$pcn2, df$covid, heading ="In-hospital",outline=TRUE)
c4 <- myperf(df1$pcnb2, df1$covid, heading ="In-hospital",outline=TRUE)


res <- rbind(c1,c2,c3,c4)


res[,4]-1.96*sqrt(abs(res[,4])*(1-abs(res[,4]))/100)
res[,4]+1.96*sqrt(abs(res[,4])*(1-abs(res[,4]))/100)

write.csv(res,"table2.csv")





tiff("fig1.tiff", height =4, width =4.5, units = 'in', res=600)
par(mfrow=c(1,1), mar=c(4,4,1.5,1.5), oma=c(1.5,2,1,1))

plot.roc(df$covid,df$carm_n,
ci=TRUE,col="black",lty=1,asp=NULL,cex.axis=0.8,grid=TRUE,ylim=c(0.025,0.975),xlim=c(0.975,0.025),grid
.col="gray",identity.lty=2)
plot.roc(df1$covid,df1$carm_nb,add=TRUE,col="brown",lty=1)
plot.roc(df$covid,df$cars_n,add=TRUE,col="darkgreen",lty=1)
plot.roc(df1$covid,df1$cars_nb,add=TRUE,col="navyblue",lty=1)
title("COVID-19")

dev.off()
```

```
tiff("fig2.tiff", height =8, width =8, units = 'in', res=600)
par(mfcol=c(2,2), mar=c(4,4,1.5,1.5), oma=c(1.5,2,1,1))

val.prob.ci(df$pcn1,df$covid, dostats = NULL, xlab="Predicted Risk",ylab="Observed Risk",xlim=c(-0.0,0.41),ylim=c(-0.15,0.41));title("CARM_N")
val.prob.ci(df1$pcnb1,df1$covid, dostats = NULL, xlab="Predicted Risk",ylab="Observed Risk",xlim=c(-0.0,0.41),ylim=c(-0.15,0.41));title("CARM_NB")
val.prob.ci(df$pcn2,df$covid, dostats = NULL, xlab="Predicted Risk",ylab="Observed Risk",xlim=c(-0.0,0.41),ylim=c(-0.15,0.41));title("CARS_N")
val.prob.ci(df1$pcnb2,df1$covid, dostats = NULL, xlab="Predicted Risk",ylab="Observed Risk",xlim=c(-0.0,0.41),ylim=c(-0.15,0.41));title("CARS_NB")

dev.off()

#####in hospital slopes
slope = summary(glm(df$covid~df$lcarm_n2c,data=df,family="binomial"))$coefficients[2,1];slope
se = summary(glm(df$covid~df$lcarm_n2c,data=df,family="binomial"))$coefficients[2,2]
ll=slope - 1.96*se;ll;ul=slope + 1.96*se;ul

slope = summary(glm(df1$covid~df1$lcarm_nb2c,data=df,family="binomial"))$coefficients[2,1];slope
se = summary(glm(df1$covid~df1$lcarm_nb2c,data=df,family="binomial"))$coefficients[2,2]
ll=slope - 1.96*se;ll;ul=slope + 1.96*se;ul

slope = summary(glm(df$covid~df$lcars_n2c,data=df,family="binomial"))$coefficients[2,1];slope
se = summary(glm(df$covid~df$lcars_n2c,data=df,family="binomial"))$coefficients[2,2]
ll=slope - 1.96*se;ll;ul=slope + 1.96*se;ul

slope = summary(glm(df1$covid~df1$lcars_nb2c,data=df,family="binomial"))$coefficients[2,1];slope
se = summary(glm(df1$covid~df1$lcars_nb2c,data=df,family="binomial"))$coefficients[2,2]
ll=slope - 1.96*se;ll;ul=slope + 1.96*se;ul
```
